# ROS as Regulators of Cellular Processes in Melanoma

**DOI:** 10.1155/2021/1208690

**Published:** 2021-10-23

**Authors:** Isabella Venza, Mario Venza, Maria Visalli, Germana Lentini, Diana Teti, Francesco Stagno d'Alcontres

**Affiliations:** ^1^Department of Biomedical, Dental, Morphological and Functional Imaging Sciences, University of Messina, Messina, Italy; ^2^Department of Clinical and Experimental Medicine, University of Messina, Messina, Italy; ^3^Department of Adult and Childhood Human Pathology, University of Messina, Messina, Italy; ^4^Scylla Biotech Srl, Messina, Italy

## Abstract

In this review, we examine the multiple roles of ROS in the pathogenesis of melanoma, focusing on signal transduction and regulation of gene expression. In recent years, different studies have analyzed the dual role of ROS in regulating the redox system, with both negative and positive consequences on human health, depending on cell concentration of these agents. High ROS levels can result from an altered balance between oxidant generation and intracellular antioxidant activity and can produce harmful effects. In contrast, low amounts of ROS are considered beneficial, since they trigger signaling pathways involved in physiological activities and programmed cell death, with protective effects against melanoma. Here, we examine these beneficial roles, which could have interesting implications in melanoma treatment.

## 1. Introduction

It has become increasingly evident in recent years that the production of reactive oxygen species (ROS) is involved in the regulation of normal cell functions and that its dysregulation can be responsible for the onset of harmful events [1]. ROS are intermediate products in reduction-oxidation (redox) reactions during conversion of O_2_ to H_2_O. They include free oxygen radicals and nonradical oxidants, as reported in Table 1 that shows the main oxygen and nitrogen reactive species. Oxygen radicals include superoxide anion (O_2_·^−^), hydroxyl (·OH), peroxyl (ROO·) and alkoxyl radicals (RO·), nitric oxide (NO·), organic radicals (R·), thiyl radicals (RS·), sulfonyl radicals (ROS·), thiylperoxyl radicals (RSOO·), and disulfides (RSSR). Nonradical oxidants comprise hydrogen peroxide (H_2_O_2_), ozone (O_3_), singlet oxygen (^1^O_2_), organic hydroperoxides (ROOH), hypochloride (HOCl), peroxynitrite (ONO^−^), nitrosoperoxycarbonate anion (ONOOCO_2_^−^), nitrocarbonate anion (O_2_NOCO_2_^−^), dinitrogen dioxide (N_2_O_2_), and nitronium (NO_2_^+^), as well as highly reactive lipid- or carbohydrate-derived carbonyl compounds [2, 3]. It should be noted that H_2_O_2_ is an oxidizing agent that is classified as ROS since it generates the hydroxyl radical ·OH.

### 1.1. Intracellular ROS Production

**Figure 1:**
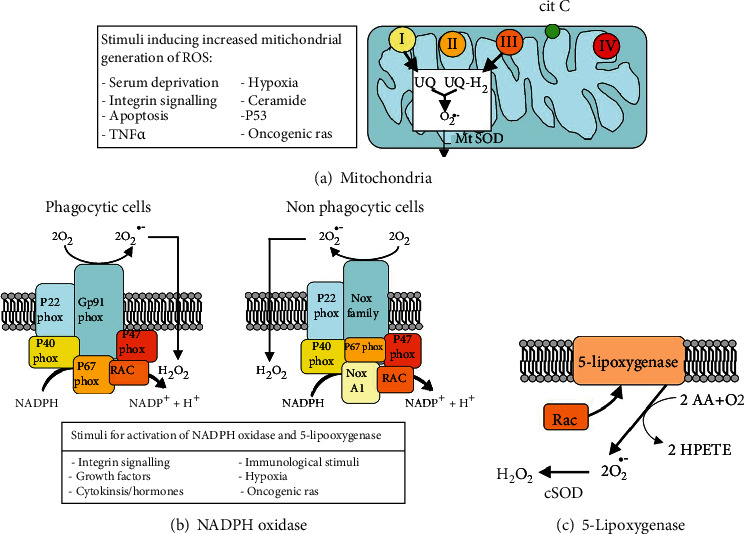
Main cellular sources of ROS. From Novo, E., Parola, M. “Redox Mechanisms in Hepatic Chronic Wound Healing and Fibrogenesis,” *Fibrogenesis Tissue Repair* 2008; 1: 5. doi: 10.1016/j.canlet.2008.02.035.

ROS may be produced by either nonenzymatic or enzymatic pathways and may originate from reactions involving organic compounds or ionizing radiations. Superoxide anion generates hydroxyl radical by interacting with hydrogen peroxide in the Haber-Weiss reaction. On the other hand, hydroxyl radical can be generated from H_2_O_2_ in the nonenzymatic Fenton reaction, in which Fe^++^or Cu^++^ act as single-electron donors [4]. Intracellular ROS can be produced at the level of mitochondria and plasma membranes. Production on membranes involves NAPH oxidase and 5-lipoxygenase enzymes (Figure 1). Multiple enzyme systems differently dislocated in the cell are involved in ROS production. Some of them are cytosolic enzymes, such as cyclooxygenases, myeloperoxidase, nitric oxide synthase (NOS), lipoxygenases, and microsomal cytochrome p450-dependent oxygenases [5, 6]. Others are restricted to various cell compartments, such as the mitochondria and cell membranes, as mentioned above.

### 1.2. Plasma Membrane

It has been known for a long time that phagocytes can produce ROS at the plasma membrane level through the activation of nicotinamide adenine dinucleotide phosphate hydrogen (NADPH) oxidase, as part of a host defense mechanism. However, it is now recognized that NADPH oxidase is also expressed in nonphagocytic cells. It is composed of membrane proteins, namely, the cytochrome b_558_, consisting of gp91*^phox^* or Nox2 and p22*^phox^*, and the small G protein Rap1A, as well as the cytosolic proteins p40*^phox^*, p47*^phox^*, p67*^phox^*, and G Rac2 (Figure 1). Upon activation, the cytosolic proteins are phosphorylated and recruited to the plasma membrane [7–11]. In this way, the transfer of electrons leads to the direct reduction of free oxygen. NADPH oxidases homologous to Nox2 have been classified into the following 3 groups, on the basis of their evolutionary relationships: (1) the group closest to Nox2, which includes Nox1, Nox3, and Nox4; (2) the dual oxidase groups, Duox-1 and Duox-2; and (3) Nox5 [12, 13]. Nox enzymes can be classified also into the following groups, based on their regulation: (a) the group including Nox1, Nox2, and Nox3, which need cytosolic regulator proteins to exert their function; (b) Nox4, which is constitutively activated; and (c) Nox5, Duox1, and Duox2, which bind calcium through their EF-hands and are controlled by intracellular calcium levels [13].

### 1.3. Mitochondria

Mitochondria represent the main site of cellular ROS generation (about 90%). In particular, O_2_·^−^ is produced in the electron transport chain [1, 5] through the transferring of a single electron to oxygen by reduced coenzymes or prosthetic groups or xenobiotics reduced by specific enzymes [14]. Superoxide anion is released from complexes I and III at low levels into the mitochondrial matrix and in higher quantities into both sides of the inner mitochondrial membrane to easily reach their targets in the cytoplasm (Figure 1; [15]). Mitochondrial biogenesis and proper mitochondrial function are also essential to maintain physiological ROS levels and prevent the detrimental effects deriving from excessive ROS production. Such activity has great relevance in cell biology, since mitochondrial DNA is exposed to unbalanced ROS generation from both environmental and nutritive factors and is highly sensitive to oxidative damage. Initially, the mitochondrion was considered as an O_2_ sensor capable of responding to hypoxic conditions by ROS-mediated regulation of gene transcription [16]. However, mitochondria are involved in the transcriptional activity of the cell in several other ways. One of the most important for cell survival is mediated by the activation of the serine/threonine protein kinase D1 (PKD1) by mitochondrial ROS, leading to the induction of nuclear factor kB (NF-*κ*B) [17]. This represents a novel mitochondria-to-nucleus signaling pathway involved in the activation of genes expressing antioxidant proteins [18, 19]. Indeed, this can be viewed as a homeostatic mechanism, since it results in PKD-mediated detoxification from mitochondrial reactive oxygen species.

### 1.4. Peroxisomes

Peroxisomes play an important role in the dynamic spin of ROS generation and scavenging. Indeed, these organelles are involved in a respiratory pathway uncoupled from oxidative phosphorylation, in which H_2_O_2_ is generated from O_2_ reduction [20]. Moreover, peroxisomes contain several types of ROS-generating enzymes such as xanthine oxidase that produces superoxide anion in the peroxisomal matrix and membranes [21, 22], NO synthase (NOS) that is responsible of formation of NO·, as well as enzymes involved in lipid metabolism, such as Acyl-CoA oxidases that generate H_2_O_2_ [23]. H_2_O_2_ and NO· pass through the peroxisomal membranes to take part in cellular signaling pathways.

### 1.5. Cell Detoxification from ROS

**Figure 2:**
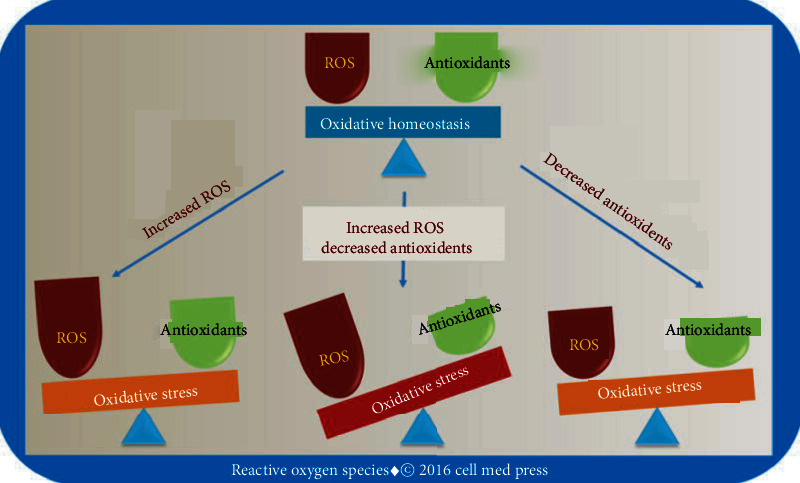
Oxidative homeostasis involves the balancing actions of ROS and antioxidants. Oxidative stress can result from excessive ROS production, decreased antioxidant activity, or both. From Li, R., Jia, Z., and Trush, M.A. “Defining ROS in Biology and Medicine,” *React Oxyg Species (Apex)*, 2016; 1(1): 9–21. doi: 10.20455/ros.2016.803.

ROS homeostasis is maintained through the balance between production and scavenging by antioxidant mechanisms used by cells to prevent excessive ROS production and accumulation. Indeed, a persistent increase of ROS levels exceeding antioxidant cell defenses, or a deficient antioxidant system, can result in a state of “oxidative stress” that provokes severe modifications to the functions of cell components (Figure 2). Moreover, diverse types of ROS are heterogeneously distributed at different concentrations in the same tissue or in different tissues [24]. Detoxification from different types of ROS is obtained by an antioxidant system including either enzymes that are contained in different cell compartments, and selectively eliminate specific ROS species, or nonenzymatic molecules [25]. Structure and functions of antioxidant enzymes may be regulated by posttranslational changes such as phosphorylation, acetylation, and oxidation of cysteine residues [26]. In particular, oxidative posttranslational modifications (Ox-PTM) represent a switch able to change protein structure and function, including the catalytic properties of enzymes [27, 28]. In this regard, the thiol (RSH) functional group of cysteine residues within proteins represents the major cellular ROS target, especially of H_2_O_2_ and superoxide anion [29]. Therefore, cysteine residues and their intermolecular disulfide bond are considered to be “sensors” that are capable to monitor the redox homeostasis in cells [30–32]. H_2_O_2_ produced in response to growth factors oxidizes the thiol functional group of the cysteine residues contained in various signaling pathway proteins playing a role in modulation of their activities [29, 32, 33]. In fact, cystein oxidation may reversibly modulate the functions of some proteins, as shown in the following examples: (i) the inactivation of the catalytic site of protein tyrosine phosphatase SHP-2 induced by high concentrations of H_2_O_2_ allows PDGF to activate MAPK signaling [33–35], (ii) the p38 mitogen-activated protein kinase (MAPK) signaling pathway is activated in association with conformational modifications of the disulfide reductase thioredoxin (Trx) that leads to the release of the complexed protein apoptosis stimulating kinase 1 (ASK1) [36, 37], (iii) HOCl regulates the activity of matrix metalloprotease 7 (MMP-7) by oxidation of cystein residues in proenzyme metalloprotease 7 *in vitro* [38], and (iv) cystein oxidation increases the activity of the cell redox sensor Ca^2+^-release channels (ryanodinereceptors) in heart and skeletal muscle *in vitro* [39, 40].

### 1.6. Antioxidant Enzymatic Systems

#### 1.6.1. Superoxide Dismutases (SODs)

Among the antioxidant enzymes, the most representative is SODs that block the excessive activity of O_2_·^−^ at the production sites [41] through the conversion of superoxide anion to H_2_O_2_ (Figure 1). SODs show high antioxidative activity although they are present at low levels in the cell. Indeed, ROS concentration is balanced by the regulation of epigenetic and posttranscriptional expression of three genes encoding in mammals for three different isoforms of SODs: copper-zinc superoxide dismutase (Cu/ZnSOD, SOD1) encoded by the *sod1* gene, manganese superoxide dismutase (MnSOD, SOD2) encoded by *sod2*, and extracellular superoxide dismutase (EC-SOD, SOD3) encoded by *sod3*. SODs differ on the basis of their molecular weight, homodimer or homotetramer forms, metal cofactors, location into the cell, or the extracellular environment [42–44]. The distribution of SOD1 activity within the cell is related with the sites of superoxide anion production, thus confining the antioxidant activity in cytoplasm [42, 45], nuclei, and lysosomes [41], as well as in the matrix of peroxisomes [46]. In particular, peroxisomes use SOD1 to directly scavenge the superoxide anion O_2_·^−^produced as an additional by-product of xanthine oxidase. A small fraction of SOD1 in the mitochondrial intermembrane space [47] has a minor role as antioxidant [41]. The superoxide anion undergoes different degradation pathways, according to the site of its production and its inability to cross the lipid barrier of membranes [48]. O_2_·^−^generated in the inner membranes is dismuted to H_2_O_2_ by SOD1, while it becomes the substrate for SOD2 in the mitochondrial matrix [3, 49–51]. Extracellular SOD3 is the only specific antioxidant enzyme that eliminates superoxide in the extracellular environment. It is present in extracellular fluids, such as plasma, lymph, and synovial fluid [52, 53], although it is also strongly expressed in a cell- and tissue-specific way in various types of connective tissues and in the walls of blood vessels at the level of the extracellular matrix and on cell membranes [54, 55], in the lung [56, 57]. Binding of SOD3 to the extracellular matrix and to cell surfaces is mediated by heparin sulfate proteoglycans [58]. Cellular up-take of SOD3 and its subsequent nuclear translocation, induced by vitamin C, defend the DNA from ROS activity and/or transcription of redox-sensitive genes [59, 60]. The protective effect of SOD3 against oxidative DNA damage was demonstrated in 17*β*-estradiol- (E2-) induced breast tumors in female rats and in MCF-10 breast epithelial cells that significantly express SOD3 after treatment with the antioxidant butylated hydroxyanisole (BHA) or vitamin C [60]. Indeed, extracellular SOD3 can translocate to the nucleus [59]. In the extracellular matrix SOD3 regulates the bioavailability of nitric oxide in vessel walls and transforms nitric oxide into peroxynitrite, a potent oxidizing agent [61]. Similarly to SOD1, the activity of SOD3 is associated with reduction and reoxidation of the copper ion at the catalytic site. The transfer of copper to specific cellular targets occurs by the transport protein Antioxidant-1 (Atox1) that could play a role in SOD3 upregulation [62].

#### 1.6.2. Catalase (CAT)

This enzyme is encoded by *CAT* gene that is involved in the synthesis of four subunits bound to heme group. CAT is located in peroxisomes and in the cytosol and acts as scavenger of hydrogen peroxide derived from SOD activity, through its conversion to water and molecular oxygen. This transformation occurs at a high rate of turnover, since 6 million molecules of H_2_O_2_ are converted per minute [63]. The reaction catalyzed by CAT includes two steps. After interaction with H_2_O_2_, CAT is transformed into an oxidized intermediate named compound I and returns in its reduced state by reacting with a second molecule of H_2_O_2_. In presence of low H_2_O_2_ concentrations and donors of one electron, compound I is transformed into an intermediate called compound II [64]. Since H_2_O_2_ is removed by different cellular enzymatic systems, catalase antioxidant activity may be different in various tissues because of its diverse expression and concentration levels [65].

#### 1.6.3. Glutathione (GSH) System

An important detoxifying mechanism occurring in the conversion of H_2_O_2_ to O_2_ is the glutathione (L-*γ*-glutamyl-L-cysteinyl-glycine) multienzyme system that includes reduced glutathione (GSH), glutathione reductase (GR), glutathione peroxidases (GPx), and glutathione S-transferases (GST). The importance of glutathione is also linked with different activities, such as transport of amino acids across the plasma membranes, regulation of transcription factors, including NFkB, or ability to act as a cofactor of numerous ROS detoxifying enzymes. GSH is an electron-donor substrate [66] occurring in the reduction of protein disulfide bonds to cysteine in oxidative stress conditions. In this way, GSH is oxidized to glutathione disulfide (GSSG) in a cytosolic coupled reaction in which GPX catalyzes a H_2_O_2_ transformation to H_2_O and O_2_ [63]. Next, a constitutive activity of GR converts GSSG to GSH in the mitochondrial matrix. GSH can also be produced by *de novo* synthesis or extracellular uptake. The GSH/GSSG ratio is an indicator of cell oxidative stress [67]. GPXs can be considered as an enzyme family originating from a common gene ancestor. They are located in the cytosol and in mithocondria and play an important role as scavengers of lipid peroxides. Expression of the GPX isoforms differs in relation to the type of tissue. *Gpx1*, *Gpx2*, and *Gpx6* genes are involved in glutathione-mediated age-related detoxification and redox mechanisms and are probably coregulated by an antioxidant response element (ARE) in the promoter region [68]. Consistent with the presence of the rare amino acid selenocysteine (SeCys) residue at the active site, GPXs are distinct in selenocysteine containing GPXs (GPX1-4, GPX6) and nonselenocysteine containing GPXs (GPX5, GPX7, GPX8), that include a Cys residue rather than SeCys [69, 70]. Among the selenocysteine-containing GPXs, GPX1 is located in various cell compartments such as cytosol, nucleus, and mitochondria and is ubiquitously expressed in all mammalian tissues, whereas GPX2 is expressed in breast and intestinal epithelial cells [71], but its substrates are unknown [69]. GPX3 is mostly synthesized in kidney cells, but it is considered as a plasma component, since it is secreted extracellularly as a result of lack of a retention signal for the endoplasmic reticulum [69]. From the blood, GPX3 binds to the basement membranes of the gastrointestinal tract, epididymis, and lung epithelia [72]. GPX3 uses various secreted thiols as reductants, such as thioredoxin and glutaredoxin, in addition to GSH as an electron donor. GPX4 (PHGPX) acts with GSH to reduce phospholipid hydroperoxides contained in membranes and lipoproteins [73]. It is present in three different isoforms in cytoplasm, mitochondria, and sperm nucleus. The cytosolic form is ubiquitously expressed, while the mitochondrial and nuclear forms are mainly expressed in testis [69]. Among GPXs that do not contain seleno-cysteine, GPX5 plays a role as a phospholipid hydroperoxidase, and it is mostly and differentially expressed in three different transcripts in mouse epididymis, where it prevents cell oxidative stress and DNA mutations [69, 74–76]. GPX7 is a nonselenocysteine-containing phospholipid hydroperoxide glutathione peroxidase (NPGPX) that is expressed in many tissues as a mitochondrial, as well as a nonmitochondrial form in the cytosol [77]. GPX7 protects against singlet oxygen-generated lipid peroxidation by removing lipid hydroperoxides from cell membranes [78, 79]. In addition, GPX7 plays an important role in oxidative stress as an endoplasmic reticulum- (ER-) resident sensor/transducer of signals in pathways involved in the regulation of ROS levels, through its interaction with target proteins [70, 80]. Furthermore, hydroperoxide reduction by GPX7, in association with ER oxidoreductin1 (Ero1) flavoproteins and GRP78 chaperone, is correlated with formation or rearrangement of disulfide bonds in oxidative protein folding mediated by protein disulfide isomerase (PDI) [81]. Indeed, GSH and PDI are both considered alternative substrates of GPX7, since the active site of GPX7 oxidizes PDI when GSH concentration is low [82]. GPX8 is a transmembrane protein located in the ER, like GPX7, with which it shares similarity in amino acid sequences and domains, the association with Ero1, and PDI peroxidase activity in disulfide bond formation with the production H_2_O_2_ [83], which is quickly eliminated by GPX8 to protect cells from oxidative stress [70, 84]. Moreover, GPX8 has been shown to be a cellular substrate of hepatitis C virus NS3-4 protease [85]. GSTs include eight classes of isoenzymes (Alpha, Mu, Pi, Theta, Kappa, Sigma, Zeta, and Omega). In addition to their detoxification role, consisting in the reduction of hydroperoxides with the formation of GSSG, or conjugation of GSH with nonsubstrate ligands in the metabolism of exogenous compounds, such as chemical carcinogens or drugs, they also play a noncatalytic activity, such as membrane transport [86, 87]. GSTs are mainly located in cytosol, but they were also detected in nucleus and in mitochondria. In particular, glutathione *S*-transferase Kappa 1 (GSTK1) is present in peroxisomes, where it plays a fundamental role in the elimination of lipid peroxides [88]. It shows a TRX-like domain analogous to that of GPX [89].

#### 1.6.4. Thioredoxin (Trx)

Trx is a cellular redox system, including Trx and Trx reductase. Trxs include three isoforms coupled with GSH/GSSG system in redox regulation: Trx1 that is located in the cytoplasm and moves into the nucleus in oxidative stress situations, where it interacts with transcription factors, whereas Trx2 is present in mitochondria, and Sp-Trx3 is spermatid-specific [90, 91]. NADPH-dependent Trx reductase catalyzes the reduction of disulfide bonds of two cysteine residues contained in the catalytic site of Trx [3]. Trx reductase is protected by calcium from nitrosoureas deactivation in metastatic melanotic and amelanotic melanomas [92].

#### 1.6.5. Peroxiredoxins (PRDXs)

PRDXs are thioredoxin peroxidases that catalyze the reduction of organic hydroperoxides, H_2_O_2_, and peroxynitrite [3, 93]. In mammalian cells, PRDXs include six isoforms that are grouped into three classes, namely, the 1-Cys (Prx VI) atypical 2-Cys (Prx V) and 2-Cys subgroups (Prxs I–IV), along with the recycle of sulfenic acid with formation of a disulfide linkage between two cysteine residues in their active site [94]. PRDXs are distributed in the cytosol, mitochondria, peroxisomes, plasma membranes, nucleus, and endoplasmic reticulum and are involved in programmed cell death and cell proliferation [2, 23, 66, 90, 94–96]. Among PRDXs, the function of PRDX1 as an antioxidant enzyme is currently debated, since it has been found to be extremely susceptible to oxidative stress [97]. In contrast, under oxidative stress conditions, an important role seems to be played by the ER-resident protein PRDXIV, which acts as H_2_O_2_ sensor for protein folding [98, 99]. However, PRDXs show a low catalytic activity compared to the efficiency of other antioxidant enzymes, such as GPXs or catalase. Despite this, PRDXs are the main scavengers of signaling ROS, and their activity is regulated by phosphorylation and sulfenylation.

#### 1.6.6. Sulfiredoxins (SRXs)

These make up a family of proteins containing a conserved cysteine residue and catalyze the reduction of sulfenylated PRDX, which reactivate them.

#### 1.6.7. Glutaredoxins (GRXs)

GRXs reduce cysteine residues in disulphide substrates and are reduced, in turn, by NADPH-dependent glutathione reductase [100].

### 1.7. Antioxidant Nonenzymatic Systems

Nonenzymatic antioxidants also play a critical role in neutralizing free radicals and oxidants inside cells, since they modify ROS to form less reactive species. They include water-soluble antioxidants that are present in cytosol, such as ascorbate, reduced GSH, which plays a role as a spontaneous antioxidant, and low molecular-weight scavengers such as coenzyme Q-10, lipoic acid, and lipid-soluble antioxidants, such as a-tocopherols, carotenoids, flavonoids, and omega-3 acids, as well as trace metals, such as selenium and zinc that can be externally supplied [101]. In addition to its role as spontaneous antioxidant, GSH can function as an electron-donor substrate in enzymatic reactions for ROS detoxification [66]. Cancer cells have developed the ability to specifically upregulate antioxidant mechanisms, in order to elude the negative effects of the high ROS levels induced by genetic changes and altered metabolism. Therefore, these homeostatic systems give cancer cells a selective advantage for their survival. This is considered an apparent paradox, since high production of ROS is associated with elevated antioxidant levels in cancer [102, 103]. Several lines of evidence indicate that ROS may act as a double-edged sword, since they function either as harmful agents or as beneficial signaling molecules. Thus, they can function as “bad” or “good” molecules depending on type, quantity, duration, and localization of specific ROS production in cell physiology [103]. On the “huggly” side, one should consider the aptitude of ROS to induce chronic diseases, including cancer [104], which is linked to the ability of excessive ROS production to produce cell or tissue damage. Alternatively, ROS produced by nonphagocytic cells may trigger a physiological response to many extracellular stimuli, whereas ROS produced by phagocytic cells defend the host against microorganisms.

### 1.8. ROS as Harmful Molecules

ROS can induce deregulation of redox-sensitive pathways and considerable damage to macromolecules, such as oxidation of proteins with enzyme inhibition, peroxidation of lipids at plasma membranes with production of aldehyde derivatives, DNA single- and double-strand breaks, base substitutions, formation of adducts [105], and chromosomal aberrations [106], as well as genomic instability, epigenetic, and gene expression modifications involving tumor suppressor genes and oncogenes [63, 107]. Moreover, ROS can stimulate signaling pathways, such as Ca^2+^-signaling, protein phosphorylation, and transcription factor activation or act as second messengers in the activation of pathways triggered by ligand-receptor interaction [108].

### 1.9. ROS as Beneficial Molecules

ROS are not only damaging for cells but are also considered critical signaling molecules in the regulation of various pathways. In fact, ROS can act as beneficial molecules, since they affect several cell functions. It is known that ROS determine a positive or negative cell response on proliferation in relation to their concentration. In breast cancer MCF-7 cells, low levels of hydrogen peroxide and increased superoxide anion concentrations are linked to reduce activity of MnSOD, leading to increased cell proliferation, while the overexpression of MnSOD leads to augmented production of H_2_O_2_ and suppression of the malignant phenotype, with inhibition of hypoxic accumulation of hypoxia-inducible factor-1 (HIF-1) and vascular endothelial growth factor (VEGF) [109]. It was reported that ROS play diverse roles on lung carcinoma H460 cells, depending on the type and quantity of specific ROS produced [110]. In particular, ·OH promotes cell motility, while O_2_·^−^ and H_2_O_2_ play an inhibitory role in cell migration and invasion. Cell migration is promoted by treatment with catalase, whereas it is inhibited by hydrogen peroxide [110].

### 1.10. Factors Affecting the Biological Functions of ROS

It is now well established that the concentration of ROS within cells plays a crucial role on cell functions [4, 102]. Low ROS levels have protective effects on normal physiological activities, serving as effectors in the immunological defense against pathogens, mediators in signaling pathways involved in various cellular processes, such as proliferation, differentiation, survival, and critical intermediates in vascular tone regulation, control of ventilation, and erythropoietin production. At subtoxic concentrations, H_2_O_2_ acts as an intracellular messenger in pathways regulated by different growth factors, such as PDGF and EGF. Increased concentrations of H_2_O_2_ inactivate protein tyrosine phosphatases (PTPs) and the lipid phosphatase PTEN, leading to accumulation of phosphorylated proteins capable of activating various signaling pathways [111]. On the contrary, at moderate or high concentrations, ROS stimulate the expression of genes reactive to stress and can produce harmful oxidative DNA damage. However, the effects of high levels of ROS depend on the cell type, since they can generate harmful consequences, such as oxidative stress, when they act on normal cells, or host-beneficial effects when the act on cancer cells, by causing their growth suppression through cell cycle inhibition and apoptosis. Therefore, in addition to the concentration of ROS, also the cell type influences the response to oxidative stress. A complex interplay occurs between ROS and various cell systems, and this must be considered in evaluating the effects of ROS on cell functions. Many factors come into play in the balance between advantageous and detrimental effects. In fact, in addition to the amount of ROS and the cell type, various conditions, including the length of oxidant production, the kind of reactive species produced, and the source of production (plasma membranes, cytosol, nucleus, mitochondria, peroxisome, endoplasmic reticulum), may contribute to trigger a physiological response or cell/tissue damage. Thus, both the type of ROS and their local concentration collectively determine whether redox signaling or oxidative stress-induced damage occurs. The location of the molecular target(s) and the associated physiological or pathological conditions should also be considered [26, 112]. These are only the most cited factors determining the cell fate. As the imbalance in ROS generation and removal is linked to the development or exacerbation of many pathological conditions, like cancer, chronic inflammation, metabolic diseases, aging, and neurodegenerative disorders, it has become increasingly clear that ROS metabolism must be kept under strict control, both in terms of quantity and quality. Therefore, a thorough understanding of such issues may help in the prevention and management of several diseases and in reverting the harmful effects of ROS in living systems.

### 1.11. Role of ROS as Messengers

A persistent increase of ROS causes modifications in gene expression and activation of cell death pathways, such as apoptosis or necrosis, thereby significantly contributing to disease onset and progression. Specificity in ROS signaling pathways is linked to sensors that are sensitive to variations in ROS cell concentration and regulate the expression of scavengers to maintain a basal ROS level [100]. As mentioned above, elevated concentrations of different kinds of ROS are responsible for cell damage, whereas moderate levels of ROS act as regulatory mediators in the activation of redox-sensitive signaling pathways in response to the action of various extracellular stimuli. These include cytokines, such as transforming growth factor *β* (TGF-*β*) [113, 114], TNF-alpha [115], and interleukin-1 b [116], as well as growth factors [116–118], such as platelet-derived growth factor (PDGF) [119] and epidermal growth factor (EGF) that trigger H_2_O_2_ generation [120]. These extracellular stimuli are in turn associated with transcriptional cell changes related to oxidative stress [121]. The specificity of ROS on diverse signaling pathways also depends on the pKa of signaling molecules, the redox status around the signaling pathway, and the total redox status in the cell [122]. In this context, both extracellular ROS, as hydrogen peroxide, or oxidants produced within the cells, as superoxide anion, may act as second messengers in signaling transduction systems and in regulating pathways sensitive to the redox status [5, 123]. Such pathways involve signaling molecules, including p38/mitogen activated protein kinases (MAPKs), phosphatidyl inositol-3 kinase (PI3K)/Akt [124, 125], various protein kinase C (PKC) family members, and c-Jun N-terminal kinase (JNK) [126, 127], that are modulated by NADPH oxidase and pathway*-*specific transcription factors such as nuclear factor-*κ*B (NF-*κ*B) [128], activator protein 1 (AP-1), hypoxia inducible factor (HIF), and p53. Only few signaling molecules and pathways have been found to be activated by mROS increase. In particular, proapoptotic signaling molecules, such as c-Jun N terminal kinase (JNK) and p38, in response to high levels of mROS induce apoptosis through caspase activation [129]. On the contrary, Akt, activated in response to exogenous ROS or growth factors, protects cells from apoptosis mediated by mROS through downregulation of Bcl-2 family members. In T lymphocytes, ROS activate two distinct pathways: superoxide anion triggers the proapoptotic FasL signal pathway, and H_2_O_2_ production regulates the proliferative signal by ERK activation [130, 131].

### 1.12. ROS and Cancer

Compared to normal cells, cancer cells are exposed to high levels of ROS, which may represent a response to increased metabolism, elevated production of growth factors and activation of cell signaling pathways [120], altered mitochondrial or peroxisomal functions, increased activity of oxidant enzymes, or increased generation of ROS in inflammation [132, 133]. On the other hand, ROS in cancer cells are involved in the regulation of signaling molecules during cell cycle progression and proliferation [134], survival, apoptosis [135], intercellular adhesion, cell motility, and angiogenesis [3]. This effect may be of particular interest, since ROS regulate signal transduction pathways during apoptosis, and antioxidants such as N-acetylcysteine, or overexpression of MnSOD can block or suspend apoptosis. An example is offered by SOD3 which regulates cell proliferation in a dose-dependent manner. In anaplastic thyroid cancer cells, high SOD3 levels are correlated with cell growth arrest through p53-p21 signaling, while decreased SOD3 mRNA expression correlates with increased malignant cell proliferation mediated by the Ras oncogene signal pathway [136, 137]. Indeed, ROS affect with different signals the transduction pathways and the expression of genes involved in cell proliferation. In this way, superoxide plays a role of second messenger in the Ras and Rac signaling pathway and is involved in uncontrolled cell transformation and proliferation [138], despite H_2_O_2_ best meets the criteria required of a second messenger such as enzymatic production and degradation [139]. The main features of cancer cells, along with melanoma and nonmelanoma skin tumors, are the generation of extracellular superoxide anion and the so-called “H_2_O_2_-catabolizing phenotype” [140–145]. The catalase activity at the membrane level prevents transformed cells from receiving signals promoting apoptosis. In contrast, interestingly, catalase inhibition activates apoptosis only in cancer cells and not in normal ones [146]. When Nox1 is silenced, the proapoptotic effect of catalase inhibition is abolished. Therefore, the malignancy is held by anion superoxide generation and H_2_O_2_ elimination, such indicating the beneficial role for H_2_O_2_ in specifically eliminating cancer cells through apoptosis. The role of the hydrogen peroxide in inducing apoptosis is currently deemed to be of considerable importance so as to promote therapeutic plans that provide for the inhibition of catalase membrane [147]. Therefore, the promalignant effects of ROS are not to be assigned to hydrogen peroxide, which activates apoptosis, but rather to the production of superoxide ion. More recent studies have highlighted the role of redox imbalance in skin carcinogenesis and its complexity, due to the multiple and interrelated pathways involved. In the skin, low levels of ROS induce physiological responses, such as wound healing and skin repair, besides dermal angiogenesis [148]. If ROS accumulation occurs in response to external stimuli or in pathological conditions, DNA damage may result in carcinogenesis and development of melanoma, spinous cell carcinoma, and basal cell carcinoma. However, if toxic levels of ROS are reached, cell death can disrupt tumor development. ROS act in several ways in inducing skin cancer: they can activate protooncogenes, such as BRAF, NRas, or suppress antioncogenes, such as p53, or modify gene expression through epigenetic alterations, or affect the related signal pathways [149]. ROS affect a series of transcription factors that are involved in carcinogenesis, such as those regulating proliferation, metastasis, angiogenesis, and inflammation. Therefore, ROS has become a privileged target in cancer therapy [150].

### 1.13. ROS in Signal Transduction with Negative Effects in Melanoma

**Figure 3:**
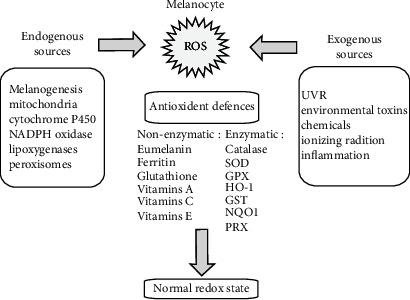
Listed are various endogenous and exogenous sources of ROS in melanocytes. ROS levels are decreased by the action of a number of antioxidants. From Denat L. et al. “Melanocytes as Instigators and Victims of Oxidative Stress,” *Journal of Investigative Dermatology*, 2014 Jun; 134(6): 1512-1518. doi: 10.1038/jid.2014.65.

**Figure 4:**
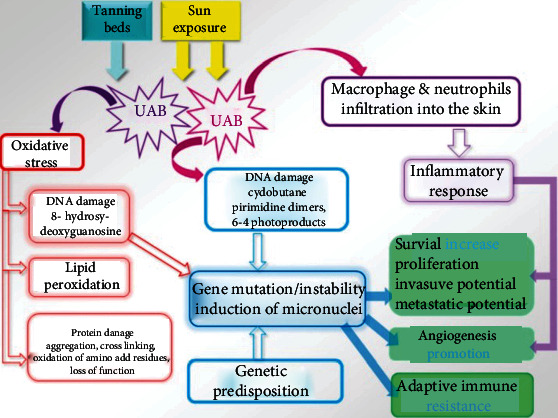
UV radiation and oxidative stress promote melanoma formation. Both sun exposure and tanning beds are sources of UV. Among these, UVA is mostly responsible for oxidative stress, causing DNA and protein damage as well as lipid peroxidation. DNA damage leads to gene instability and mutagenesis, impacting the main cellular processes involved in cancerogenesis, cancer growth, metastasis, and adaptive immune resistance. Likewise, UVB modulate those processes by the activation of inflammatory response. From Wróbel, S., Przybyło, M and Stępień, E. “The Clinical Trial Landscape for Melanoma Therapies,” *J. Clin. Med*. 2019, 8(3): 368. doi: 10.3390/jcm8030368.

Although ROS have been considered just as harmful agents in the past, recent evidence supports their crucial role as regulator molecules in various signaling pathways, whose effects are context-dependent. An altered redox status occurs in melanoma development through the activation of different signal transduction pathways mediated by NADPH oxidase family members (including NOX 1-5 and DUOX1/2) and other ROS-producing molecules.  Figure 3 depicts the balance between ROS production and detoxification in melanocytes. Different studies report changes in expression levels of proteins involved in oxidant mechanisms, but the exact molecular mechanisms responsible for ROS-dependent promotion of melanoma development have not yet been fully elucidated. NOXs/DUOXs are responsible for maintaining optimal cellular levels of ROS. Tumor cells may produce high levels of ROS that are the result of deregulation of NOXs present either in melanoma (NOX1 and NOX4) or in various other types of tumors, such as prostate cancer (NOX1 and NOX5) and glioblastoma (NOX4) [151]. *Nox1* and *Nox4* are overexpressed in melanoma cell lines, including metastatic cells. In particular, in the melanoma cell line Wm3211 the overexpression of *Nox1* correlated with increased cell invasion. Nox4 mediates melanoma proliferation by regulating G2-M cell cycle progression, and modifications in Nox4 expression are present in the early stages of melanoma development [152]. Interestingly, expression profiles for Nox4 show that proliferating normal epithelial melanocytes highly express only Nox4 and its associated subunit p22*^phox^*, while gp91*^phox^* (Nox2) is only slightly expressed. In contrast, some human melanoma cells strongly express the NADPH oxidase components p22*^phox^* and gp91*^phox^* in plasma membrane and the cytosolic p67*^phox^*, whereas p47*^phox^* expression level is low [153]. In B16 mouse melanoma cells, Nox4 is expressed in large quantities compared to other isoforms. Nox4 and ROS production are increased by the *α*-melanocyte-stimulating hormone (*α*-MSH), *via* the microphthalmia-associated transcription factor (MITF), which induces Nox4 gene expression [154]. Since melanin synthesis induces the silencing of *Nox4* gene by increasing tyrosinase gene expression, ROS generation could be also inversely correlated with melanin formation via a negative feedback mechanism of regulation that may be altered in skin pathologies [154]. Upregulation of Nox1 is an early event in melanoma transformation [155] and *Nox1* overexpression regulates melanoma invasion through upregulation of matrix metalloproteinase-2 [156]. Inhibition of Nox1 activity blocks migration of melanoma cells [157]. NADPH oxidases are responsible for the costitutive activation of the transcription factor NFkB by malignant melanoma cells which in turn results in increased cell proliferation. NADPH oxidase inhibitors reduce proliferation. In particular, the NADPH oxidase inhibitor diphenylene iodonium (DPI) inhibits the costitutive DNA binding of transcription factors to NFkB and cAMP-response elements, thus suggesting a crucial role of NADPH oxidase in melanoma proliferation [153] and of antioxidant therapy for the interruption of oxidant signaling in melanoma [158]. This assumption is confirmed by more recent studies showing that some enzymes involved in the regulation of redox status, such as peroxiredoxins (Prx) I and Prx II, are downregulated in melanoma as compared to dysplastic and benign naevi. Moreover, sulfiredoxin and Prx IV expression apparently showed a protective role in melanoma and was associated with a better prognosis [159]. The complex interplay between UV radiation and oxidative stress leading to melanoma is shown in Figure 4.

A first mechanism by which excessive ROS production can promote melanoma formation and development is simply *via* direct induction of DNA damage and mutagenesis. ROS, however, may also favor the development of melanoma and other tumors by modulating the activation of signaling pathways and transcription factors, such as NF-*κΒ*. For example, ROS-induced oxidation of LC8, a multifunctional protein of the dynein motor complex, can increase NF-*κΒ* activation. This is linked to the ability of reduced LC8 to bind to the NF-*κ*B component I-*κ*B*α* in a redox-dependent manner, blocking its phosphorylation by IKK. ROS-oxidized LC8 dissociates from I-*κ*B*α*, leading to NF-*κ*B activation [160]. NF-*κ*B activation, in turn, could favor melanoma progression through its antiapoptotic effects and by creating an inflammatory microenvironment [161]. ROS, however, may also inhibit NF-*κ*B activation *via* oxidation of thioredoxin, a protein that, when present in the nucleus in a reduced state, increases NF-*κ*B activity by promoting its binding to DNA [160]. Moreover, prolonged oxidative stress may lead to direct oxidation of NF-*κ*B heterodimers and reduced DNA binding. Thus, while acute oxidative stress leads to increased NF-*κ*B activation, sustained ROS production may have more complex effects.

### 1.14. ROS as Beneficial Molecules in Melanoma

ROS can be considered beneficial molecules in melanoma as they may, under certain conditions, activate programmed cell death. In association with selected inhibitors of cell growth, ROS may be mediators in signal pathways leading to apoptosis. The proapoptotic antitumor antibiotic DC-81-enediyne induces death of human melanoma A375 cells by the involvement of ROS, caspase-3 activation, PARP degradation, and activation of the p38/MAPK and AP-1 signaling pathways [162]. Moreover, curcumin activates apoptosis in the same cell line [163]. Treatment with Parthenolide too induces apoptosis through ROS generation, leading to depletion of proteinthiols and glutathione (GSH) and dissipation of the mitochondrial membrane potential (Dcm), with condensation and fragmentation of chromatin and activation of caspase-independent and AIF-mediated apoptosis in melanoma cells [164]. Cytokine melanoma differentiation associated gene 7- (*mda*-7-) induced apoptosis in melanoma cells is mediated by ROS that induce significant decrease in both BCL-2 and BCL-XL and upregulation of BAX and BAK [165]. The generation of ROS is the signal pathway triggered by Benzofuroxan N-Br and N-I derivatives to induce cytoxicity and inhibition of AKT activation in melanoma B16F10-Nex2 cells [166]. A similar mechanism is employed by Spatane diterpinoids isolated from the brown marine algae *Stoechospermum marginatum* that they were shown to induce apoptosis in B16F10 melanoma cells in a concentration-dependent manner through ROS generation. The resulting oxidative stress induced an imbalance in Bax/Bcl-2 ratio that disrupted the inner mitochondrial transmembrane potential (*ΔΨ*m) resulting in cytochrome c redistribution to the cytoplasm and activation of caspase-mediated apoptotic pathway. Moreover, apoptosis was reached also through another signaling pathway involving the deregulation of PI3K/AKT. Such effects were also shown in C57BL/6 mice bearing B16F10 melanoma. Spatane diterpinoid from the brown algae, *Stoechospermum marginatum*, induces apoptosis via ROS-induced mitochondrial-mediated caspase dependent pathway in murine B16F10 melanoma cells [167]. Another mechanism employed by ROS as antimelanoma agents was shown by studies reporting that Simvastatin, a prooxidant agent responsible for an increased amount of intracellular ROS and overexpression of catalase and peroxiredoxin-1, is able to induce senescence in human melanoma cells by activation of p53/p21 pathway [168]. A similar effect is exerted by Nexrutine that increases the constitutively elevated oxidative stress in melanoma cells to inhibit their survival mediated by PI3K/AKT/mTOR [169]. Photodynamic therapy-induced ROS increase has been shown to significantly reduce melanoma proliferation through cell autophagy mechanism, such supporting the notion that the oxidative stress is responsible of melanoma cell damaging [170]. Several compounds are able to induce apoptosis in melanoma cells through ROS generation by either mitochondria-dependent [171] or mitochondria-independent pathways [172]. The former mechanism is engaged by cerium (Ce) oxide nanoparticles (CNP; nanoceria) which selectively kills A375 melanoma cells through the increase of ROS concentration, prevalently hydrogen peroxide, at mitochondrial level. Such event occurs concomitantly to mitochondrial thiol oxidation and is followed by modifications in mitochondrial bioenergetics, dynamics, and cristae morphology, and ultimately by mitochondrial dysfunction-induced cell death [173]. Moreover, increased concentrations of ROS can reduce melanoma development through activation of cell cycle regulators and arrest of cell cycle in G2/M phase by the inhibition of Cdc25c and cyclin A [171]. Recently, several small molecule ROS inducers have been employed with the aim to pharmacologically elevate intracellular levels of ROS through various mechanisms and to target and disrupt tumor cells in a selective way [174]. For example, atmospheric gas plasmas (AGP) are able to upregulate intracellular ROS and to induce apoptosis in melanoma, but not in normal melanocyte, cells by oxidative stress-induced activation of the TNF-ASK1-JNK/p38–caspase-3/7 apoptotic pathway [175]. More recently, it has been shown that VB1, a compound purified from the seed of the Chinese herb *Vitex negundo*, inhibits melanoma cell proliferation and promotes apoptosis by increasing ROS levels, thereby causing DNA damage and cell death. The effect was selective for melanoma cells, including BRAF inhibitor-resistant cells [176]. Similar effects were exerted by the novel calchone derivative lj-1-59 that, by inducing ROS elevation, blocks melanoma cell proliferation at the G2/M phase and triggers different apoptosis pathways [177].

**Figure 5:**
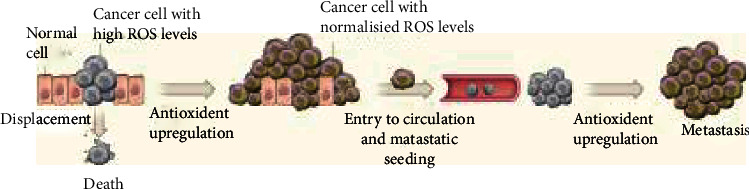
High metabolic activity can lead to increased ROS levels and cell death in melanoma and other cancer cells. Conversely, effective antioxidant responses can result in cell growth and metastasis. From Harris, I., Brugge, J. “The Enemy of My Enemy Is My Friend”. *Nature* 2015 12 (11); 527 (7577): 170-1. doi:10.1038/nature15644, commentary to Piskounova E, Agathocleous M, Murphy MM, Hu Z, Huddlestun SE, Zhao Z, Leitch AM, Johnson TM, DeBerardinis RJ, Morrison SJ. “Oxidative Stress Inhibits Distant Metastasis by Human Melanoma Cells.” *Nature*. 2015 Nov 12; 527 (7577): 186-91. doi:10.1038/nature15726. Epub 2015 Oct 14.

Treatment of melanoma through the combined effects of two different ROS inducers has been successfully achieved employing low-dose UVA irradiation and brusatol (BR), a quassinoid isolated from *Brucea javanica* plant. Both cause ROS accumulation, the former one by endogenous photosensitization [178], the latter one by deregulating nuclear factor E2-related factor 2 (Nrf2), a transcription factor belonging to the cap “n”collar family of leucine-zipper (b-ZIP) proteins. Following oxidative stress, Nrf2 is activated and induces the expression of genes, such as heme oxygenase 1 (HO-1), NAD(P)H:quinone oxidoreductase-1 (NQO1), and glutathione S-transferase (GST) drug transporters to restore homoeostasis [179]. On the contrary, it was clearly shown that inhibition of the Nrf2-mediated antioxidant defense system sensitizes cancer cell to therapy [180]. Indeed, it was reported that BR is a potent inhibitor of Nrf2 activation, and that in such a way, it can reduce tumor proliferation and cancer chemoresistance [181]. More recently, it was shown that, in A375 melanoma cells, cotreatment with (UVA + BR) either inhibited melanoma cell growth and proliferation both in vitro and in vivo and/or activated cell apoptosis. The UVA-induced increase in of ROS was further enhanced by the BR-mediated caused reduction of Nrf2 expression with a consequent inhibition of AKT signaling. Therefore, cotreatment of UVA and BR reduced melanoma development by blocking AKT-Nrf2 cascades [182]. These data have promoted studies showing that the excess of antioxidants is detrimental in melanoma treatment as it causes melanoma cell metastasis (Figure 5).

### 1.15. ROS and Epigenetic Pathways in Melanoma

In the last decades, several evidences pointed to the role of epigenetics in cancer onset and progression as key factor involved in tumorigenesis, even though cancer was generally considered to be the resultant of genetic mutation accumulation [183]. Really, it has been clearly shown that also the genetic view regards changes in gene expression prevalent in cancer [183–185]. Closer studies of the epigenetic regulatory mechanisms showed that a gene expression state is determined not only at the transcriptional level but even more at posttranscriptional level. A complex scenario emerged where the simple genetic concept that a mutation in oncogenes or suppressor genes led to cancer by an abnormal or reduced expression, respectively, is now substituted by a variety of mechanisms involving either gene chemical modifications or an intricate network of regulatory RNAs.

The genetic path to cancer is relatively straightforward: mutation of tumor suppressors and/or oncogenes causes either loss or gain of function and abnormal expression. The epigenetic pathway to cancer is more complex and is determined by changes in chromatin structure, including DNA methylation, histone variants and histone modifications, and nucleosome remodeling, as well as small noncoding regulatory RNAs [186]. During tumor initiation and progression, the epigenome goes through multiple alterations, including a genome-wide loss of DNA methylation (hypomethylation), frequent increases in promoter methylation of CpG islands, changes in nucleosome occupancy, and modification profiles. It is now believed that oxidative stress may affect the so-called epigenetic machinery, since an emerging role of ROS as inducers of epigenetic changes has been established. To understand the molecular mechanisms underlying the redox balance, the use of epi-drugs that have been available in the last few years proved to be crucial. They can take part in epigenetic processes by the reactions of nucleophilic substitution.

ROS are active intermediaries in either DNA methylation or histone chemical modifications, but they have been also implicated in the regulation of microRNA (miRNA) pathways, by altering mRNA stability and their transport inside the cytosol. ROS may induce an aberrant hypermethylation by increasing the expression of DNA methyltrasferases (DNMTs). The methylation of CpG islands in the promoter of oncosuppressor genes leads to gene silencing and tumor onset [187]. On the other hands, ROS are also able to induce a global hypomethylation of the genome [149] and, intriguingly, the hypomethylation of histone H3K9 leads to melanoma epigenetic instability [188]. ROS may interfere with gene expression by affecting also the histone acetylation/deacetylation level by their activity on acetyltransferase (HAT) or histone deacetylase (HDAC). Gene expression alterations caused by ROS-induced histone modification depend either on the amino acid residues involved or the level of histone acetylation [189]. It is generally believed that histone deacetylase (HDACi) inhibitors may trigger cancer cell death through ROS generation [190, 191]. In this context, the histone deacetylase inhibitor vorinostat, which is well known to induce 8-oxo-G, a marker of oxidative DNA damage [192], was shown to dramatically increase ROS levels only in BRAF (V600E) mutant melanomas, which acquired resistance to MAPK inhibitors. This type of melanoma already displayed elevated levels of ROS that were further increased by vorinostat treatment. In such a way, a very significant tumor regression was observed, as consequence of the toxic elevation of cell ROS. Interestingly, the vorinostat effect was BRAF (V600E) mutant and MAPK inhibitors-resistant melanoma specific [193]. Among the HDACs involved in cancer development, HDAC6 has caught the attention of many scholars, and recently, it has been shown to be related to melanoma onset [194]. Previous studies have shown that histone deacetylase 6 (HDAC6) plays critical roles in many cellular processes related to cancer. However, its biological roles in the development of melanoma remain unexplored. Our aim was to investigate whether HDAC6 has a biological role in human melanoma development and to understand its underlying mechanism. In the present study, HDAC6 expression was upregulated in melanoma tissues and cell lines. Knockdown of HDAC6 significantly inhibited the proliferation and colony formation ability of A375.S2 cells, promoted cell arrest at G0/G1 phase and apoptosis. Additionally, western blotting analysis showed that HDAC6 silencing suppressed Bcl-2 and enhanced Bax levels, activated caspase-9 and caspase-3, further activated the release of cytochrome c from mitochondria into the cytoplasm, and finally induced apoptosis involving the mitochondrial pathway. Knockdown of HDAC6 triggered a significant generation of ROS and disruption of mitochondrial membrane potential (MMP). Furthermore, the ROS inhibitor NAC reduced HDAC6 siRNA-induced ROS production and blocked HDAC6 siRNA-induced loss of MMP and apoptosis. NAC also significantly blocked HDAC6 siRNA-induced mtDNA copy number decrease and mitochondrial biogenesis and degradation imbalance. In conclusion, the results showed that knockdown of HDAC6 induced apoptosis in human melanoma A375.S2 cells through a ROS-dependent mitochondrial pathway. Interplay between ROS signaling and miRNA pathway was described as possible cause of cancer, since both are dysregulated in this pathology [195]. But not only that, in several types of cancer, including skin cancer, there is a reciprocal relationship between ROS and miRNA profiles. Indeed, miRNAs may be regulated, induced, or repressed, by ROS as well as by hypoxia, or in turn are themselves capable of inducing ROS increase [196]. miRNAs are greatly implicated in the regulation of oxidative stress by interacting with the nuclear factor erythroid 2-related factor 2 (Nrf2), a transcription factor that controls the expression of several genes involved in the response to oxidative stress. As some miRNAs modify the expression of genes responsible either for ROS production or antioxidant response, they have been defined “redoximiRs” [197]. Recent studies have evidenced the double way in which ROS and miRNA interact and the underlying mechanisms [196]. A microRNA profiling analysis revealed that exposure to H_2_O_2_ modifies the set of microRNA contents through epigenetic modifications, such as alteration of the methylation status of miRNA genes or biogenesis. In such a way, the oxidative stress may interfere with carcinogenesis not only by DNA damage-induced mutations but also through epigenetic alterations of miRNA genes. Similarly, miRNAs are capable of regulating intracellular ROS levels by targeting enzyme involved in ROS generation and elimination [198]. Dysregulation of miR-125b following ROS exposure has been involved in skin carcinogenesis via interfering with the expression of genes involved in cell proliferation [198]. Many other miRNAs silencing the expression of oncogenes able to induce cell proliferation have been shown to be hypermethylated by the oxidative stress in melanoma, such as miR-34b, miR-34c, miR-148, and miR-9. Thereby, their targets *MYC* and cyclin-dependent kinase 6 resulted to be overexpressed leading to cell transformation [199]. Recently, special attention was paid to the complex network of long noncoding RNAs (lncRNA), miRNAs, and mRNAS, named “competing endogenous RNA (ceRNA)” that regulates gene expression in cancer cells by posttranscriptional mechanisms, as firstly described by Salmena et al. [200]. lncRNAs are longer than 200 nucleotides and are unable to exert protein-coding activity. They function as sponges towards miRNA, preventing them from blocking gene expression [201]. According to the hypothesis of Salmena et al., lncRNAs, by affecting mRNA expression through the interaction with miRNA response elements, widen genetic information, and if an alteration occurs in the network equilibrium, it may have great implications in cancer pathogenesis. Recently, therapeutic interventions based on lncRNA analysis improved prognosis and quality of life in melanoma patients in early stages of disease or with Breslow thickness less than 2 mm [202]. Six lncRNAs (AL050303, LINC00707, LINC01324, RP11-85G21, RP4-794I6.4, and RP5-855F16) have been shown to affect MAPK pathways, immune and inflammatory responses, and focal adhesion pathways, thereby suggesting that they may be significant in the prognosis of patients with melanoma [203]. More recently, a signature of seven lncRNAs has been implicated in melanoma metastasis, as they are differentially expressed in primary and metastatic melanoma and are independently related to overall survival [204]. Among the identified seven lncRNAs, myocardial infarction associated transcript (MIAT) aroused great interest and significance as prognostic marker in melanoma. It was shown to be expressed in patients with a better prognosis, and its potential role in improving immune response was evidenced in light of a greater sensitivity to immunotherapy. Interestingly, H19 that was found to be dysregulated in metastatic melanoma, as compared with primary melanoma, and to be associated with a poor diagnosis constitutes an integral part of the hypoxia, p53, and cancer pathway [205]. Several lncRNAs have been correlated with an increase of RO and shown to be regulators of cardiovascular diseases related to hypoxia, cardiotoxicity, and ischemia-reperfusion [206]. Therefore, ROS act not only in signal transduction pathways but also interfere with the competing endogenous RNA network in regulating gene expression. In this context, many efforts are aimed at employing lncRNA-based therapies in ROS-related diseases. For example, knockdown of the lcnRNA growth arrest-specific transcript 5 (GAS5) increases the levels of superoxide anion and oxidized glutathiones by altering the redox balance in melanoma cells. In such a way, reduced GAS5 expression is linked to progression of melanoma [207]. It is now well established that ROS are crucial mediators of signaling cascades regulating cell proliferation, invasion, migration, and apoptosis and that they are able to activate or suppress important cell functions. A fine interplay exists between ROS and noncoding RNAs, in which the former are regulated by noncoding RNAs and, in turn, the expression of the latter is modified by ROS-induced alterations of proteins responsible for noncoding RNA transcription and maturation [208]. However, although considerable evidence points to the involvement of ROS in the expression of coding and noncoding RNA in cancer, much remains to be known about this in melanoma.

## 2. Concluding Remarks

One of the main causes of melanoma onset is exposure to ultraviolet rays, which induce the production of significant concentrations of ROS. However, in this neoplastic condition, ROS represent much more than secondary products of the redox processes induced by ultraviolet rays. The concentration, the production site, and the specific type of ROS determine their activities and roles, influence their involvement in almost all cellular processes, and outline their benefits and harms. It is now well known that ROS are a double-edged blade or two sides of the same coin, since in certain conditions they can be detrimental to the cell, while in others they are an integral and necessary part in signal conduction pathways. Moreover, recent evidence indicates that ROS also play an increasingly important role in the regulation of gene expression. They interfere not only with the processes of DNA methylation and histone acetylation/deacetylation but also with the increasingly complex RNA network, including mRNA, miRNA, and ceRNA, although in melanoma the relationships between ROS and ceRNA have not yet been defined. Such crucial involvement of ROS in key mechanisms of cellular functions suggests that they will represent useful targets for therapeutic approaches in melanoma.

## Figures and Tables

**Table 1:** Main oxygen and nitrogen reactive species involved in cell function.

Name	Formula	Characteristics
Hyperoxide/superoxide	·O_2_^−^	Highly unstable, signaling function, synaptic plasticity
Hydrogen peroxide	H_2_O_2_	Cell toxicity, signaling function, generation of other ROS
Hydroxyl radical	·OH	Free radical, highly unstable, very reactive reagent
Alkoxyl radical	RO·	Free radical, reaction production of lipids
Peroxyl radical	ROO·	Free radical, reaction production of lipids
Hypochlorite anion	OCl^−^	Reactive oxygen species, reactive chlorine species, enzymatically generated by mieloperoxidase
Singlet oxygen	^1^O_2_	Induced/excited oxygen molecule, radical and nonradical form
Ozone	O_3_	Environmental toxin
Nitric oxide	·NO	Environmental toxin, endogenous signal molecule
Peroxynitrite	ONOO^−^	Highly reactive reaction intermediate of ·O_2_ and ·NO
Nitrogen dioxide	·NO_2_	Highly reactive radical, environmental toxin
Nitrogen oxides	NO_x_	Environmental toxins, including NO and ·NO_2_, derived from the combustion process

RNS: reactive nitrogen species; ROS: reactive oxygen species.
